# Scalable and Transferable Reinforcement Learning for Multi-Agent Mixed Cooperative–Competitive Environments Based on Hierarchical Graph Attention

**DOI:** 10.3390/e24040563

**Published:** 2022-04-18

**Authors:** Yining Chen, Guanghua Song, Zhenhui Ye, Xiaohong Jiang

**Affiliations:** 1 School of Aeronautics and Astronautics, Zhejiang University, Hangzhou 310027, China; ch19930611@zju.edu.cn; 2 College of Computer Science and Technology, Zhejiang University, Hangzhou 310027, China; zhenhuiye@zju.edu.cn (Z.Y.); jiangxh@zju.edu.cn (X.J.)

**Keywords:** multi-agent, deep reinforcement learning, partial observability

## Abstract

Most previous studies on multi-agent systems aim to coordinate agents to achieve a common goal, but the lack of scalability and transferability prevents them from being applied to large-scale multi-agent tasks. To deal with these limitations, we propose a deep reinforcement learning (DRL) based multi-agent coordination control method for mixed cooperative–competitive environments. To improve scalability and transferability when applying in large-scale multi-agent systems, we construct inter-agent communication and use hierarchical graph attention networks (HGAT) to process the local observations of agents and received messages from neighbors. We also adopt the gated recurrent units (GRU) to address the partial observability issue by recording historical information. The simulation results based on a cooperative task and a competitive task not only show the superiority of our method, but also indicate the scalability and transferability of our method in various scale tasks.

## 1. Introduction

The last few years witnessed the rapid development of the multi-agent system. Due to its ability to solve complex computing or coordinating problems [1], it has been widely used in different fields, such as computer networks [2,3], robotics [4,5], etc. In the multi-agent system, agents try to learn their policies and execute tasks collaboratively, in either cooperative or competitive environments, by making autonomous decisions. However, in large-scale multi-agent systems, partial observability, scalability, and transferability are three important issues to be addressed for developing efficient and effective multi-agent coordination methods. Firstly, it is impossible for agents to learn their policies from the global state of the environment, as it contains massive information about a large number of agents. Therefore, they need to communicate with other agents in some ways, to reach consensus on decision making. Secondly, the previous methods either learn a policy to control all agents [6] or train their policies individually [7], which is difficult to be extended for large-scale agents. Thirdly, in existing deep-learning-based methods, the policies are trained and tested under the same number of agents, making them untransferable to different scales.

In this paper, we propose a scalable and transferable multi-agent coordination control method, based on deep reinforcement learning (DRL) and hierarchical graph attention networks (HGAT) [8], for mixed cooperative-competitive environments. By regarding the whole system as a graph, HGAT helps agents extract the relationships among different groups of entities in their observations and learn to selectively pay attention to them, which brings high scalability when applying in large-scale multi-agent systems. We enforce inter-agent communication to share agents’ local observations with their neighbors and process the received messages through HGAT; therefore, agents can reach consensus by learning from their local observations and information aggregated from the neighbors. Moreover, we introduce the gated recurrent unit (GRU) [9] into our method to record the historical information of agents and utilize it when determining actions, which optimizes the policies under partial observability. We also apply parameter sharing to make our method transferable. Compared with previous works, our method achieves a better performance in mixed cooperative–competitive environments while acquiring high scalability and transferability.

The rest of this paper is organized as follows. In Section 2, we review the related works. We describe some background knowledge of multi-agent reinforcement learning and hierarchical graph attention networks in Section 3. In Section 4, we describe a cooperative scenario, UAV recon and a competitive scenario, predator-prey. We present the mechanism of our method in Section 5. The simulation results are shown in Section 6. We discuss advantages of our method in Section 7 and draw the conclusion in Section 8. The list of abbreviations is shown in Abbreviations.

## 2. Related Work

Multi-agent coordination has been studied extensively in recent years and implemented in various frameworks, including heuristic algorithms and reinforcement learning (RL) algorithms. In [10], the authors presented a solution to the mission planning problems in multi-agent systems. They encoded the assignments of tasks as alleles and applied the genetic algorithm (GA) for optimization. The authors of [11] designed a control method for the multi-UAV cooperative search-attack mission. UAVs employ ant colony optimization (ACO) to perceive surrounding pheromones and plan flyable paths to search and attack fixed threats. The authors of [12] focused on the dynamic cooperative cleaners problem [13], and presented a decentralized algorithm named “sweep” to coordinate several agents to cover an expanding region of grids. It was also used to navigate myopic robots who cannot communicate with each other [14]. In [15], the authors designed a randomized search heuristic (RSH) algorithm to solve the coverage path planning problem in multi-UAV search and rescue tasks, where the search area is transformed into a graph. The authors of [16] proposed a centralized method to navigate UAVs for crowd surveillance. They regarded the multi-agent system as a single agent and improved its Quality of Service (QoS) by using an on-policy RL algorithm state-action-reward-state-action (SARSA) [17]. Ref. [18] proposed a distributed task allocation method based on Q-learning [19] for cooperative spectrum sharing in robot networks, where each robot maximizes the total utility of the system by updating its local Q-table.

However, as the scale of multi-agent systems increases, the environment becomes more complex while the action space of the whole system expands exponentially. It is difficult for heuristic algorithms and the original RL methods to coordinate agents since they need more time and storage space to optimize their policies. Combining deep neural networks (DNNs) and RL algorithms, deep reinforcement learning (DRL) is widely used for multi-agent coordination in cooperative or competitive environments. It extracts features from the environment state with DNN and uses them to determine actions for agents, which brings better performance. Moreover, since the environment is affected by the action of all agents in multi-agent systems, it is hard for adversarial deep RL [20] to train another policy to generate possible disturbances from all agents. Semi-supervised RL [21] also fails to apply in multi-agent systems, as it cannot learn to evaluate the contribution of each agent from the global state and their actions. DRL can either control the whole multi-agent system by a centralized policy (such as [6]) or control agents individually in a distributed framework called multi-agent reinforcement learning (MARL). In a large-scale environment, MARL is more robust and reliable than the centralized methods because each agent can train its policies to focus on its local observation instead of learning from the global state.

The goal of MARL is to derive decentralized policies for agents and impose a consensus to conduct a collaborative task. To achieve this, the multi-agent deep deterministic policy gradient (MADDPG) [22] and counterfactual multi-agent (COMA) [23] construct a centralized critic to train decentralized actors by augmenting it with extra information about other agents, such as observations and actions. Compared with independent learning [24], which only uses local information, MADDPG and COMA can derive better policies in a non-stationary environment. However, it is difficult for these approaches to be applied in a large-scale multi-agent system, as they directly use the global state or all observations when training. Multi-actor-attention-critic (MAAC) [25] applies the attention mechanism to improve scalability by quantifying the importance of each agent through the attention weights. Deep graph network (DGN) [26] regards the multi-agent system as a graph and employs a graph convolutional network (GCN) with shared weight to process information from neighboring nodes, which also brings high scalability. Ref. [8] proposed a scalable and transferable model, named the hierarchical graph attention-based multi-agent actor-critic (HAMA). It clusters all agents into different groups according to prior knowledge and constructs HGAT to extract the inter-agent relationships in each group of agents and inter-group relationships among groups, aggregating them into high-dimensional vectors. By using MADDPG with shared parameters to process those vectors and determine actions, HAMA can coordinate agents better than the original MADDPG and MAAC when executing cooperative and competitive tasks.

Various MARL-based methods have recently been proposed for multi-agent coordination. Ref. [27] designed a distributed method to provide long-term communication coverage by navigating several UAV mobile base stations (UAV-MBSs) through MADDPG. Ref. [7] presented a MADDPG-based approach that jointly optimizes the trajectory of UAVs to achieve secure communications, which also enhanced the critic with the attention mechanism, such as [25]. The authors of [28] adopted GCN to solve the problem for large-scale multi-robot control. Ref. [29] separated the search problem in indoor environments into high-level planning and low-level action. It applied trust region policy optimization (TRPO) [30] as the global and local planners to handle the control at different levels. In our previous work, we proposed the deep recurrent graph network (DRGN) [31], a novel method that is designed for navigation in a large-scale multi-agent system. It constructs inter-agent communication based on a graph attention network (GAT) [32] and applies GRU to recall the long-term historical information of agents. By utilizing extra information from neighbors and memories, DRGN performs better than DQN and MAAC when navigating a large-scale UAV-MBS swarm to provide communication services for targets that are randomly distributed on the ground.

The difference between our method and the previous works are summarized as follows. DRGN represents the observation as a pixel map of the observable area and processes it by DNN. Our method regards the global state as a graph where the nodes represent the entities in the environment and employs HGAT to process the observation. It is more effective for our method to learn relationships between agents and entities through HGAT. Moreover, our method spends less space to store the observation than DRGN, as the scale of the observation in our method is independent of the observation range. In HAMA, each agent observes up to K nearest neighboring entities per type, where K is a constant. Our method considers that agents can observe all entities inside the observation range and uses an adjacency matrix to denote the relationships of the observation, which can describe the actual observation of agents more accurately than HAMA. In addition, our method introduces GRU and HGAT-based inter-agent communication to provide extra information for agents, so they can optimize policies for coordination by learning from historical information and neighbors.

## 3. Background

### 3.1. Multi-Agent Reinforcement Learning (MARL)

The process of MARL is regarded as a decentralized partially observable Markov decision process (Dec-POMDP) [33]. In MARL, each agent *i* observes the environment state *s* and obtains a local observation $o_{i}$. Then, it selects an action according to its policy $\pi_{i}$. The environment executes the joint actions $a=(a_{1},\cdots ,a_{N})$ and transforms *s* to the next state $s^{\prime }$. After execution, each agent acquires a reward $r_{i}=R_{i}\left(s,a\right)$ and a next observation $o_{i}^{\prime }$ from the environment. Each agent aims to optimize its policy to maximize its total expected return $R_{i}=\sum_{t=0}^{T}\gamma^{t}r_{i}\left(t\right)$, where *T* is a final timeslot, and γ∈[0,1] is the discount factor.

Q-learning [19] and policy gradient [34] are two popular RL methods. The idea of Q-learning is to estimate an state-action value function Q(s,a)=E[R] and select the optimal action to maximize Q(·). Deep Q-network (DQN) [35], a Q-learning-based algorithm, uses a DNN as a function approximator and trains it by minimizing the loss:

(1) $$\begin{array}{c} L\left(\theta \right)=E_{s,a,r,s^{\prime }}\left[{\left(y-Q\left(s,a|\theta \right)\right)}^{2}\right] \end{array}$$

where θ is the parameter of the DNN. The target value *y* is defined as $y=r+\gamma \max_{a^{\prime }}Q^{\prime }\left(s^{\prime },a^{\prime }\right)$ [35], where $Q^{\prime }$ is the target network, whose parameters are periodically updated from θ. DQN also applies a replay buffer to stabilize learning.

Policy gradient directly optimizes the policy π to maximize $J\left(\theta^{\pi }\right)=E\left[R\right]$ and updates parameters based on the gradient [34]:

(2) $$\begin{array}{c} \nabla_{\theta^{\pi }}J\left(\theta^{\pi }\right)=E_{s∼p^{\pi },a∼\pi }\left[\nabla_{\theta^{\pi }}\log\pi \left(a|s,\theta^{\pi }\right)Q\left(s,a\right)\right] \end{array}$$

where $p^{\pi }$ is the state distribution. Q(s,a) can be estimated by samples [36] or a function approximator, such as DQN, which leads to the actor–critic algorithm [37].

### 3.2. Hierarchical Graph Attention Network (HGAT)

HGAT is an effective method for processing hierarchically structured data represented as a graph and introduced into MARL to extract the relationships among agents. By stacking multiple GATs hierarchically, HGAT firstly aggregates embedding vectors $e_{ij}^{l}$ from neighboring agents in each group *l* into ${e^{\prime }}_{i}^{l}$ and subsequently aggregates ${e^{\prime }}_{i}^{l}$ from all groups into ${e^{\prime }}_{i}$. The aggregated embedding vector ${e^{\prime }}_{i}$ represents the hierarchical relationships among different groups of neighbors.

## 4. System Model and Problem Statement

In this section, we describe the settings of a multi-agent cooperative scenario, UAV recon and a competitive scenario, predator-prey.

### 4.1. UAV Recon

**Figure 1:**
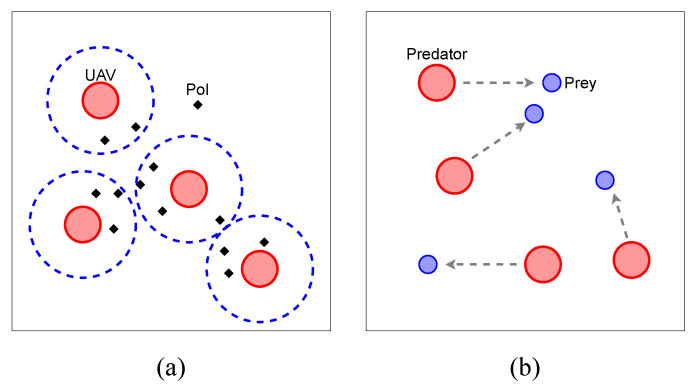
Illustrations of (**a**) *UAV Recon* and (**b**) *Predator-Prey*.

As shown in Figure 1a, we deploy *N* UAVs into a hot-spot area to scout *n* point-of-interests (PoIs) for *T* timeslots, where PoIs are randomly distributed. As we consider our UAVs to move at the same altitude, the area of our mission is two-dimensional. Each UAV has a circled recon area whose radius is considered as a recon range. If the Euclidean distance between a UAV and a PoI is less than the recon range, we consider the PoI to be covered.

In the beginning, each UAV is deployed in a random position. At each timeslot *t*, each UAV *i* determines its acceleration $acc_{i}\in \left\{(acc,0),(-acc,0),(0,acc),(0,-acc),(0,0)\right\}$ as its action. The action space of *i* is discrete. The energy consumption of *i* is defined as:

(3) $$\begin{array}{c} E_{i}=E_{h}+\frac{v_{i}}{v_{max}}E_{m} \end{array}$$

where $v_{i}$ is the velocity of *i* and $v_{max}$ is the maximum velocity of UAVs. $E_{h}$ and $E_{m}$ are the energy consumption for hovering and movement, respectively.

In our scenario, our goals of UAVs are to cover more PoIs more fairly with less energy consumption. To evaluate the quality of tasks, we consider three metrics: coverage *C*, fairness *F*, and energy consumption *E*. The score of *C* denotes the proportion of covered PoIs, which is defined as:

(4) $$\begin{array}{c} C=\frac{n_{C}\left(t\right)}{n} \end{array}$$

where $n_{C}\left(t\right)$ is the number of covered PoIs at timeslot *t*.

The score of fairness denotes how fair all PoIs are covered. Here, we use Jain’s fairness index [38] to define the score of *F* as:

(5) $$\begin{array}{c} F=\frac{{\left(\sum_{j=1}^{n}c_{j}\right)}^{2}}{n\sum_{j=1}^{n}c_{j}^{2}} \end{array}$$

while $c_{j}$ is the coverage time of PoI *j*.

Finally, UAVs need to control energy consumption in tasks. We define the score of *E* as:

(6) $$\begin{array}{c} E=\frac{1}{N}\sum_{i=1}^{N}E_{i} \end{array}$$

When executing recon missions, each UAV needs to observe local states of other UAVs and PoIs to determine its action. The local state of UAV *i* is defined as $s_{i}=\left(P_{i},v_{i}\right)$, where $P_{i}$ and $v_{i}$ are the position and the velocity of *i*, respectively. Each PoI *j*’s local state $s_{j}=\left(P_{j}\right)$. If a PoI is in UAV *i*’s observation range, we consider the PoI is observed by *i*. If another UAV *j* is in *i*’s communication range, we consider *i* can communicate with *j*. To train UAV’s policy, we define a heuristic reward $r_{i}$ as:

(7) $$\begin{array}{c} r_{i}=\frac{\eta_{1}\times r_{indv}+\eta_{2}\times r_{shared}}{E_{i}}-p_{i} \end{array}$$

where $p_{i}$ is a penalty factor. When UAV *i* flies across the border, it is penalized by $p_{i}$. $r_{indv}=-1$ if no PoIs is covered by *i* individually, otherwise $r_{indv}=n_{indv}$, where $n_{indv}$ means the number of PoIs that are only covered by *i*. $r_{shared}=0$ if *i* does not share PoIs with others, otherwise $r_{shared}=\frac{n_{shared}}{N_{share}}$, where $n_{shared}$ denotes the number of PoIs which are covered by $N_{share}$ neighboring UAVs. $\eta_{1}$ and $\eta_{2}$ are the importance factor of $r_{individual}$ and $r_{shared}$, respectively. We empirically set $\eta_{1}\gg \eta_{2}$ to encourage UAVs to cover more PoIs and avoid overlapping with others.

### 4.2. Predator-Prey

As shown in Figure 1b, we deploy $N_{predator}$ predators to eliminate $N_{prey}$ prey.

Both of them are controlled by a DRL-based method. If the distance between a predator and a prey is less than predators’ attack range, we consider the prey to be eliminated. The goal of the predators is to eliminate all prey, while the goal of the prey is to escape from the predators. The speed of the predators is slower than the prey speed, so they need to cooperate with each other when chasing prey.

The action space of the predators and the prey is the same as the UAVs in the UAV recon scenario. The local state of each predator or prey is defined as $s_{i}=\left(P_{i},v_{i}\right)$, where $P_{i}$ and $v_{i}$ are the position and the velocity of a predator or prey, respectively. We consider that each predator and prey can observe the local state of adversaries inside its observation range, while it can communicate with companions inside its communication range. The eliminated preys can neither be observed nor communicate with others. To evaluate the performance of predators and prey, we define the score as:

(8) $$\begin{array}{c} S=\frac{T-T_{eliminate}}{T} \end{array}$$

where *T* is the total timeslots of an episode, while $T_{eliminate}$ is the timeslot when all prey are eliminated.

When predator *i* eliminates prey *j*, *i* will obtain a positive reward, while *j* will obtain a negative reward. When all prey are eliminated, the predators will get an additional reward.

## 5. HGAT-Based Multi-Agent Coordination Control Method

To achieve the goals of two scenarios described in Section IV, we present a multi-agent coordination control method based on HGAT for mixed cooperative–competitive environments. In our method, the global state of the environment is regarded as a graph, containing the local state of agents and the relationship among them. Each agent summarizes the information from the environment by HGAT and subsequently computes the Q-value and action in a value-based or actor–critic framework.

### 5.1. HGAT-Based Observation Aggregation and Inter-Agent Communication

**Figure 2:**
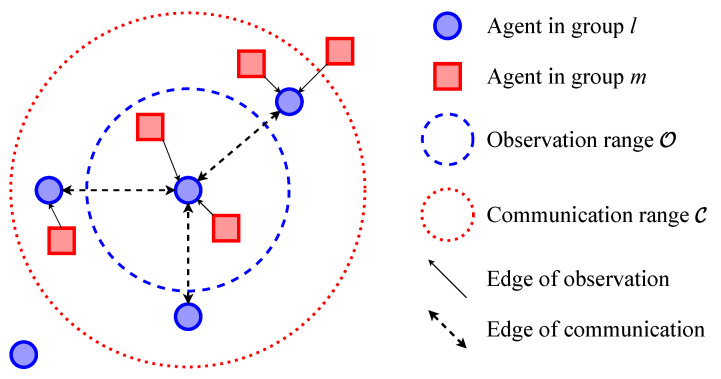
The clustering of agents and their topology.

In the multi-agent system, the environment involves multiple kinds of entities, including agents, PoIs, etc. As they are heterogeneous, agents need to treat their local states and model their relationships separately. Thus, we categorize all entities into different groups at the first step of execution in cooperative or competitive scenarios. As shown in Figure 2, *M* entities (containing *N* agents) are clustered into *K* groups and represent the environment’s state as graphs. The agents construct an observation graph $G^{O}$ and a communication graph $G^{C}$ respectively based on their observation ranges $O_{1},\cdots ,O_{N}$ and communication ranges $C_{1},\cdots ,C_{N}$. The edges of $G^{O}$ represent that the entities can be observed by agents, while the edges of $G^{C}$ represent that two of the agents can communicate with each other. The adjacency matrix of $G^{O}$ and $G^{C}$ are $A^{O}$ and $A^{C}$, respectively. *i*’s observation is defined as $o_{i}=\left\{s_{j}|j\in O_{i}\right\}$. Its received messages from the others is $m_{i}=\left\{m_{ji}|j\in C_{i}\right\}$, where $s_{j}$ is agent *j*’s local state and $m_{ji}$ is the message that *j* sends to *i*.

**Figure 3:**
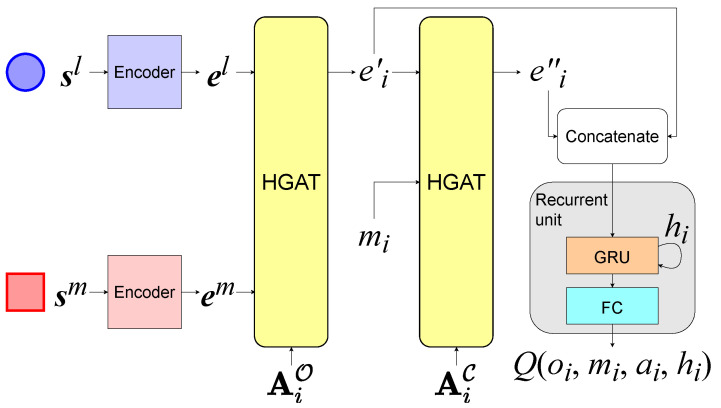
The overall structure of the network. $s^{l}$ and $e^{l}$ represent the local states and embedding vectors of agents in group *l*. $A_{i}$ denotes the *i*th row of A.

**Figure 4:**
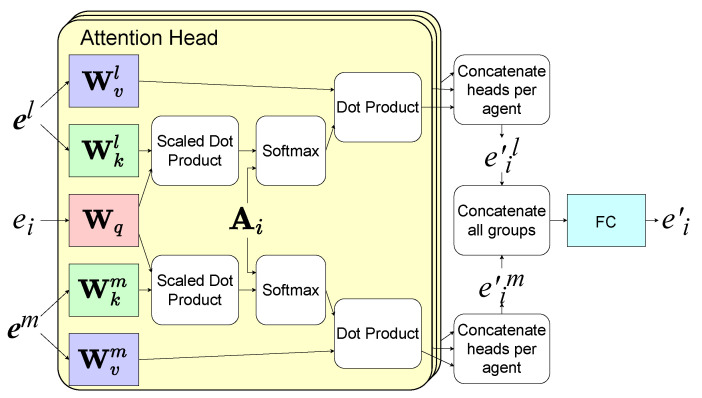
The architecture of an HGAT layer.

At each timeslot, the agents use the network shown in Figure 3 to determine their actions according to s, $A^{O}$, and $A^{C}$ received from the environment, where $s=(s_{1},,\cdots ,s_{M})$. The parameters of the network are shared among the agents in the same group. The network contains three components, a set of encoders, two stacked HGAT layers, and a recurrent unit, which consists of a gated recurrent unit (GRU) layer and a fully connected layer. GRU is a variant of the recurrent neural network (RNN). To summarize the information in each agent *i*’s observation $o_{i}$, the first HGAT layer processes $o_{i}$ into a high-dimensional aggregated embedding vector $e_{i}^{\prime }$ as shown in Figure 4. Firstly, the encoder which consists of a fully connected layer transforms the local states from each group *l* into embedding vectors as $e_{j}=f_{e}^{l}\left(s_{j}\right)$, where $f_{e}^{l}$ means the encoder for group *l*. $e_{j}$ is the embedding vector for entity *j* in group *l*. Then, it aggregates $e_{j}$ as ${e^{\prime }}_{i}^{l}=\sum_{j}\alpha_{ij}W_{v}^{l}e_{j}$[32], where $W_{v}^{l}$ is a matrix that transforms $e_{j}$ into a “value”. The attention weight $\alpha_{ij}$ represents the importance of the embedding vector $e_{j}$ from *j* to *i*, which is calculated by softmax as $\alpha_{ij}\propto \exp\left({e_{j}}^{T}{W_{k}^{l}}^{T}W_{q}e_{i}\right)$ [32] if $a_{i,j}^{O}$ in $A^{O}$ is 1, otherwise $\alpha_{ij}=0$. $W_{k}$ and $W_{q}$ transform a embedding vector into a “key” and a “query”, respectively. $A^{O}$ is used for selection so that only the local states from $O_{i}$ are summarized. To improve the performance, we use the multiple attention heads here. Finally, ${e^{\prime }}_{i}^{l}$ from all groups are aggregated into $e_{i}^{\prime }$ by a fully connected layer $f_{G}$, as:

(9) $$\begin{array}{c} e_{i}^{\prime }=f_{G}\left({\|}_{l=1}^{K}{e^{\prime }}_{i}^{l}\right) \end{array}$$

where ‖ represents the concatenation operation. We do not apply another GAT for aggregating, such as HAMA, as our approach has less computing overhead.

After calculating $e_{i}^{\prime }$, agent *i* sends it as a message $m_{ij}$ to each neighboring agent *j* in C(i). Inter-agent communication helps agents to share their observations with neighbors, which brings a better performance in coordination. To summarize each agent *i*’s received messages $m_{i}$, the second HGAT layer processes $m_{i}$ and aggregates it into another embedding vector $e_{i}^{\prime \prime }$ by the same means as shown in Figure 4. The adjacency matrix used here is $A^{C}$ instead of $A^{O}$. Our method is capable of inner-group and inter-group communication and can easily extend to a multi-hop by stacking new HGAT layers.

### 5.2. Implementation in a Value-Based Framework

This implement is based on DQN. Each agent *i* maintains hidden states $h_{i}$ for the recurrent unit and calculates its Q-values by a Q-network, as shown in Figure 3. Similar to DQN, our method also employs a target network with the same structure.

We introduce the skip-connection strategy by concatenating $e_{i}^{\prime }$ and $e_{i}^{\prime \prime }$ as an input of the recurrent unit when computing the Q-value, so agents can use the information both from their observation and others’. The Q-value is calculated as:

(10) $$\begin{array}{c} Q^{l}\left(o_{i},m_{i},a_{i},h_{i}\right)\approx f_{R}^{l}\left(e_{i}^{\prime },e_{i}^{\prime \prime },h_{i}\right) \end{array}$$

where $Q^{l}$ represents the Q-network of group *l* where *i* belongs, $f_{R}^{l}$ means the recurrent unit in $Q^{l}$, and $a_{i}$ is the action determined by *i* according to Q-values. We apply ϵ-greedy policy [35] to balance the exploitation and exploration as:

(11) $$\begin{array}{c} a_{i}=\left\{\begin{array}{cc} \arg\max_{a\in A_{i}}Q^{l}\left(o_{i},m_{i},a,h_{i}\right), & with\;probability\;1-\epsilon \\ random(A_{i}), & with\;probability\;\epsilon \end{array}\right. \end{array}$$

where $A_{i}$ is the action space of *i*.

After executing the joint actions $a=(a_{1},\cdots ,a_{N})$, the environment transforms the current state to the next and sends the next local states $s^{\prime }$, the next adjacency matrix ${A^{\prime }}^{O}$ and ${A^{\prime }}^{C}$, and the reward $r_{i}$ to each agent *i*. The experience $\left(s,A^{O},A^{C},a,r,s^{\prime },{A^{\prime }}^{O},{A^{\prime }}^{C},h,h^{\prime }\right)$ is stored in a shared replay buffer *B*, where $r=(r_{1},\cdots ,r_{N})$, $h=(h_{1},\cdots ,h_{N})$, and $h^{\prime }=\left(h_{1}^{\prime },\cdots ,h_{N}^{\prime }\right)$. $h_{i}^{\prime }$ is the next hidden state that the Q-network outputs when agent *i* calculates Q-values. $h_{i}$ is initialized to zero at the beginning of an episode.

To training the Q-network of each group, we sample *H* experiences from *B* as a minibatch and minimize the loss:

(12) $$\begin{array}{c} L\left(\theta_{l}^{Q}\right)=\frac{1}{N_{l}}\sum_{i=1}^{N_{l}}E\left[{\left(y_{i}-Q^{l}\left(o_{i},m_{i},a_{i},h_{i}|\theta_{l}^{Q}\right)\right)}^{2}\right] \end{array}$$

where $N_{l}$ means the number of agents in group *l* and $\theta_{l}^{Q}$ denote the parameters of $Q^{l}$. $y_{i}$ is the target value that calculated by the target network ${Q^{\prime }}^{l}$, as:

(13) $$\begin{array}{c} y_{i}=r_{i}+\gamma \max_{a^{\prime }\in A_{i}}{Q^{\prime }}^{l}\left(o_{i}^{\prime },m_{i}^{\prime },a^{\prime },h_{i}^{\prime }|\theta_{l}^{Q^{\prime }}\right) \end{array}$$

where $o_{i}^{\prime }$ and $m_{i}^{\prime }$ are *i*’s next observation and next received messages, respectively. $\theta_{l}^{Q^{\prime }}$ denote the parameters of ${Q^{\prime }}^{l}$, which are periodically updated from $\theta_{l}^{Q}$.

### 5.3. Implementation in an Actor–Critic Framework

Our method can also be implemented on the actor–critic framework. In this implementation, each agent *i* has an actor network and a critic network, maintaining hidden states $h_{i}^{\pi }$ and $h_{i}^{Q}$. After obtaining s, $A^{O}$ and $A^{C}$, agent *i* in group *l* computes the probability of actions as:

(14) $$\begin{array}{c} \pi^{l}\left(o_{i},m_{i},h_{i}^{\pi }\right)\approx f_{R}^{\pi^{l}}\left({e^{\prime }}_{i}^{\pi },{e^{\prime \prime }}_{i}^{\pi },h_{i}^{\pi }\right) \end{array}$$

where $\pi^{l}$ represents the actor network of group *l* and $f_{R}^{\pi^{l}}$ represents the recurrent unit in $\pi^{l}$. We employ the ϵ-categorical policy here. Agent *i* determines an action based on $\pi^{l}\left(o_{i},m_{i},h_{i}^{\pi }\right)$ with probability 1−ϵ and makes a random choice with probability ϵ. The critic network $Q^{l}$ subsequently calculates Q-values, such as the value-based framework. The hidden states $h_{i}^{\pi }$ and $h_{i}^{Q}$ and the next hidden states ${h^{\prime }}_{i}^{\pi }$ and ${h^{\prime }}_{i}^{Q}$ are stored in the replay buffer, where ${h^{\prime }}_{i}^{\pi }$ and ${h^{\prime }}_{i}^{Q}$ are the outputs of $\pi^{l}$ and $Q^{l}$, respectively.

The critic network of each group is trained by minimizing the loss $L(\theta_{l}^{Q})$, which is computed as (13). As the actor–critic framework selects actions according to $\pi^{l}\left(o_{i},m_{i},h_{i}^{\pi }\right)$ instead of the maximum Q-value, we use the expectation of the next state’s Q-value to calculate the target value $y_{i}$ as:

(15) $$\begin{array}{c} y_{i}=r_{i}+\gamma \sum_{a^{\prime }\in A_{i}}{\pi^{\prime }}^{l}\left(a^{\prime }|o_{i}^{\prime },m_{i}^{\prime },{h^{\prime }}_{i}^{\pi },\theta_{l}^{\pi^{\prime }}\right){Q^{\prime }}^{l}\left(o_{i}^{\prime },m_{i}^{\prime },a^{\prime },{h^{\prime }}_{i}^{Q}|\theta_{l}^{Q^{\prime }}\right) \end{array}$$

where $\theta_{l}^{\pi^{\prime }}$ and $\theta_{l}^{Q^{\prime }}$ are the parameters of target network ${\pi^{\prime }}^{l}$ and ${Q^{\prime }}^{l}$, respectively.

The actor network of each group is trained according to the gradient:

(16) $$\begin{array}{c} \nabla_{\theta_{l}^{\pi }}\left(J\left(\theta_{l}^{\pi }\right)\right)=\frac{1}{N_{l}}\sum_{i=1}^{N_{l}}E\left[\log\pi^{l}\left(a_{i}|o_{i},m_{i},h_{i}^{\pi },\theta_{l}^{\pi }\right)\left(Q^{l}\left(o_{i},m_{i},a_{i},h_{i}^{Q}|\theta_{l}^{Q}\right)-b_{i}\right)\right] \end{array}$$

where the baseline $b_{i}$ is designed to reduce variance and stabilize training [39], which is defined as:

(17) $$\begin{array}{c} b_{i}=\sum_{a\in A_{i}}\pi^{l}\left(a|o_{i},m_{i},h_{i}^{\pi },\theta_{l}^{\pi }\right)Q^{l}\left(o_{i},m_{i},a,h_{i}^{Q}|\theta_{l}^{Q}\right) \end{array}$$

After training, $\theta_{l}^{\pi^{\prime }}$ and $\theta_{l}^{Q^{\prime }}$ are updated as $\theta_{l}^{\pi^{\prime }}\leftarrow \tau \theta_{l}^{\pi }+\left(1-\tau \right)\theta_{l}^{\pi^{\prime }}$, and $\theta_{l}^{Q^{\prime }}\leftarrow \tau \theta_{l}^{Q}+\left(1-\tau \right)\theta_{l}^{Q^{\prime }}$, respectively [40].

Our method can be extended to continuous action space by estimating the expectation of $b_{i}$ with Monte Carlo samples or a learnable state value function $V(o_{i},m_{i})$ [23].

## 6. Simulation

### 6.1. Set Up

To evaluate the performance of our method, we conduct a series of simulations on an Ubuntu 18.04 server with 2 NVIDIA RTX 3080 GPUs. We implement a value-based (VB) version and an actor–critic (AC) version of our method in PyTorch. Each fully connected layer and GRU layer contains 256 units. The activation functions in encoders and HGAT layers are ReLU [41]. The number of attention heads is 4. Empirically, we set the learning rate of the optimizer to 0.001, and the discount factor γ to 0.95. The replay buffer size is 50 K and the size of a minibatch is 128. ϵ is set to 0.3. For the value-based version, The target networks are updated every five training steps. For the actor–critic version, we set τ to 0.01. The networks are trained every 100 timeslots and update their parameters four times in a training step.

We compare our method with four MARL baselines, including DGN, DQN, HAMA, and MADDPG. For non-HGAT-based approaches, each agent concatenates all local states in its observation into a vector, while padding 0 for unobserved entities. The parameters of networks are shared among agents in all baselines except MADDPG. We use the Gumbel-Softmax reparameterization trick [42] in HAMA and MADDPG to make them trainable in discrete action spaces. DGN is based on our proposed algorithm [31], which applies a GAT layer for inter-agent communication. We train our method and each baseline for 100 K episodes and test them for 10 K episodes.

### 6.2. UAV Recon

As summarized in Table 1, we deploy several UAVs in a 200 × 200 area where 120 PoIs are distributed. The penalty factor *p* in (7) is set to 1. We evaluate the performance of our method in the test stage under different number of UAVs and compare it with baselines.

**Figure 5:**
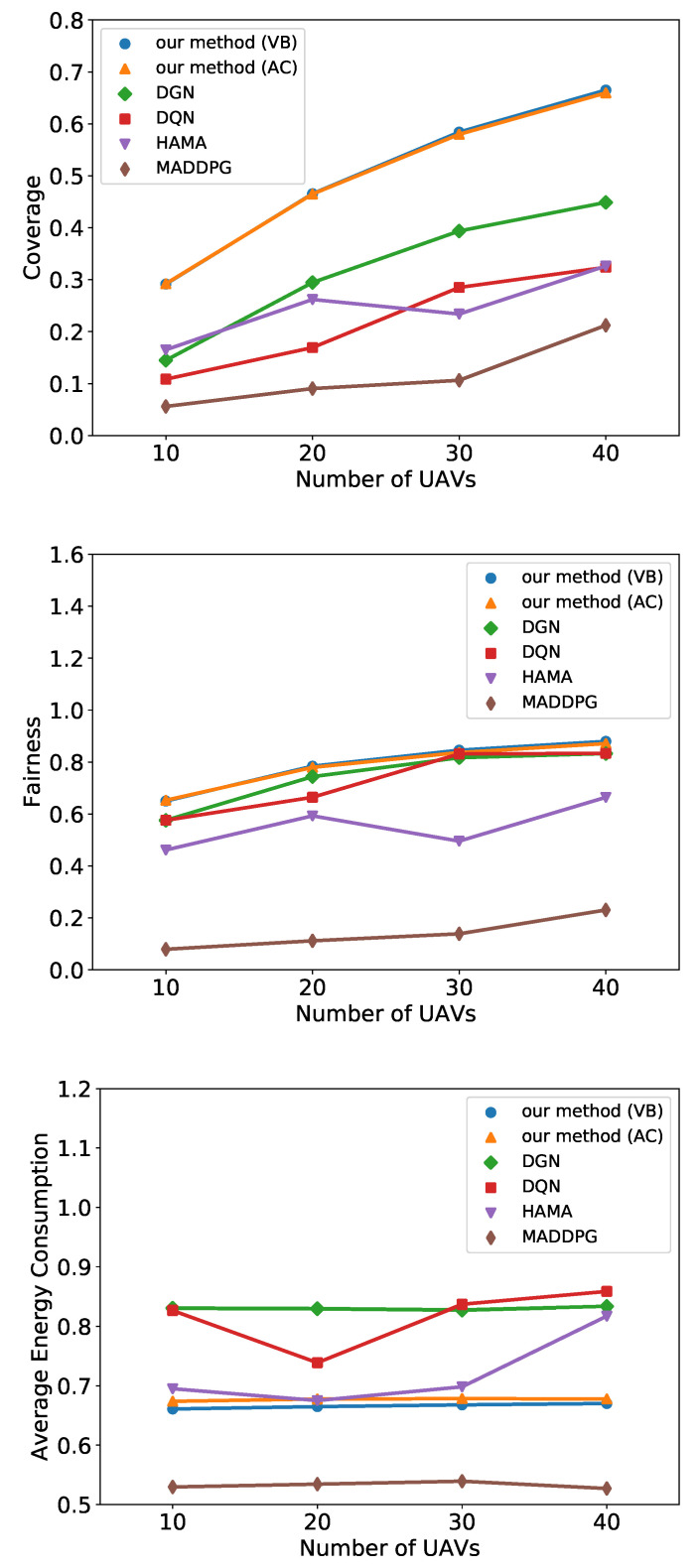
Simulation results of all methods on coverage, fairness, and energy consumption under different number of UAVs.

Figure 5 shows the performance of each method in terms of coverage, fairness, and energy consumption under different numbers of UAVs. Note that both two versions of our method are trained with 20 UAVs and transferred to a different scale of UAV swarms. From Figure 5a,b, we observe that our method outperforms all baselines in terms of coverage and fairness. Compared with DGN and DQN, our method employs HGAT to extract features from observation, which is more effective than processing raw observation vectors directly. Therefore, our method helps UAVs to search PoIs and better optimize their flight trajectories. Although HAMA also applies HGAT, UAVs cannot cooperate as effectively as our method, owing to the lack of communication. In our method, the UAVs communicate with others and process received messages by another HGAT layer. Furthermore, the recurrent unit helps UAVs to learn from the hidden states, which induces a better performance. In MADDPG, each UAV trains an individual network and concatenates observations and actions of all agents into a high-dimensional vector as an input of the critic. As the networks in MADDPG expands exponentially to the scale of the agents, it is hard to be trained effectively and efficiently in large-scale multi-agent systems. As a consequence, the MADDPG consumes more time to train but obtains the lowest score.

Figure 5c indicates that our method consumes less energy than DGN and DQN. As their flight trajectories are better, UAVs can cover more PoIs fairly while consuming less energy. The energy consumption of HAMA is considerable with our method in low-scale environments and increases when the number of UAVs is up to 40. MADDPG fails to improve coverage and fairness, so it tends to save on energy to maximize its reward.

**Figure 6:**
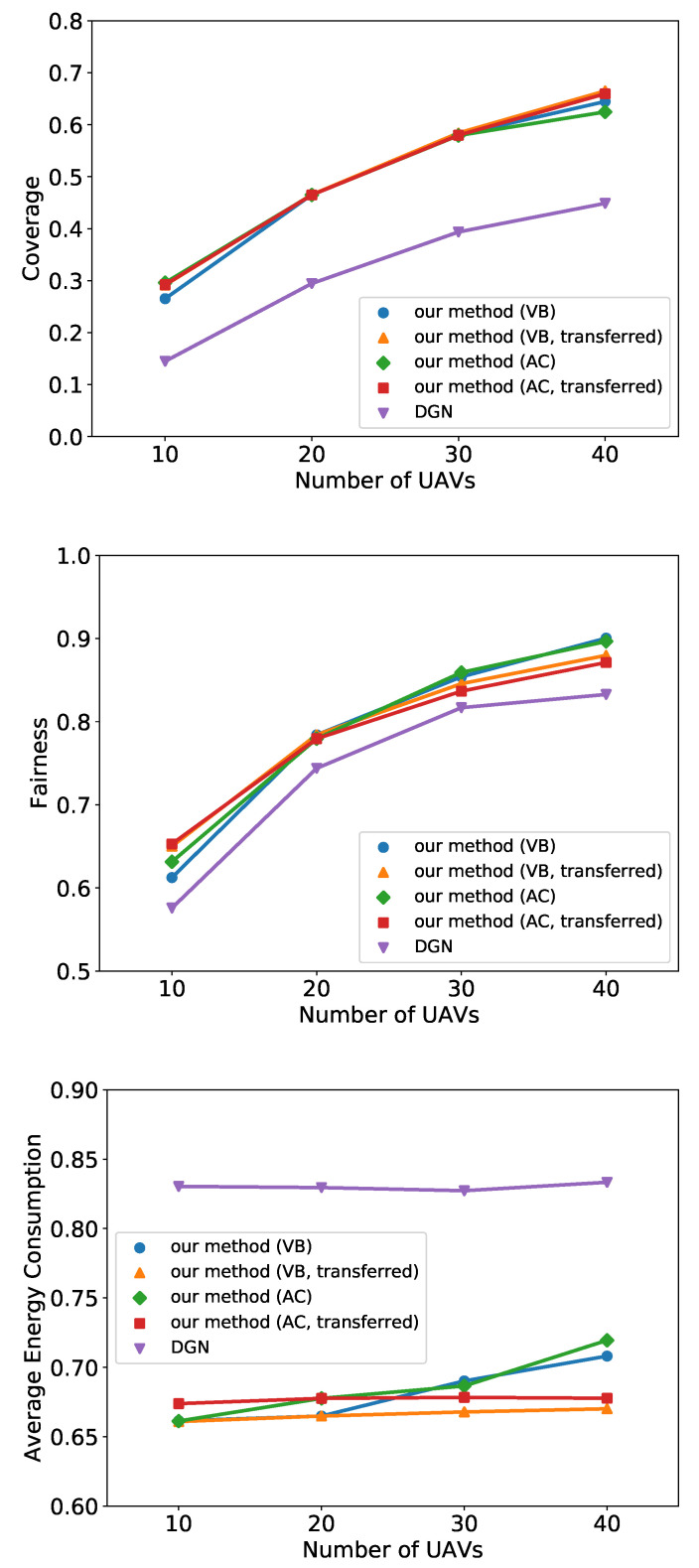
Simulation results of transfer learning on coverage, fairness, and energy consumption under different number of UAVs.

To test the capability of transferred learning, we compare the transferred policies with those trained under the same settings of testing. As shown in Figure 6, the performance does not deteriorate when the policy is transferred to execute with 10, 30, or 40 UAVs, which indicates that our method is highly transferable under various numbers of UAVs.

### 6.3. Predator-Prey

As summarized in Table 2, we deploy five predators in a 100 × 100 area to eliminate five prey. We set the attack reward of predators and prey to 10 and −10, respectively. The additional reward is set as $r_{additional}=10\times S$. We train the policy by the value-based version of our method and test it by competing with other policies trained by different methods.

Table 3 indicates that our method shows its superiority over all baselines in both roles of predator and prey. By introducing GRU and inter-agent communication, the predators obtain more information from hidden states and neighbors to decide which prey to capture. It is more flexible for predators to determine whether to chase prey individually or cooperatively. Similarly, GRU and inter-agent communication also bring more information to prey, so they can choose from various strategies to survive. For example, prey can escape from predators by their faster speed or sacrifice one of them to distract predators.

## 7. Discussion

The experimental results indicate that the performance of our method is superior to those of others in both cooperative and competitive scenarios. We assume that three components, including HGAT, GRU, and inter-agent communication, are the key factors which induce the success of our method. To validate our hypothesis, we conduct an ablation study in Appendix A to clarify the necessity of each component (HGAT, GRU, and inter-agent communication).

From Table A1, we observe a significant deterioration in performance when removing HGAT or GRU, while disabling inter-agent communication also induces a decrease in terms of coverage and fairness. To explain the necessity of each component, we assume the following reasons. Firstly, HGAT plays an important role in summarizing observations. Not only does it process the local states from all groups, but it also quantifies their importance with attention weights. In addition, HGAT models the hierarchical relationships among agents as a graph, which is effective for them to optimize their policies in dynamic environments. Secondly, GRU makes a significant contribution to overcoming the limitation of partial observability. When determining actions, GRU helps agents to remember the historical information recorded in the hidden states, such as the position of the observed PoIs in the UAV recon. It is beneficial for agents to improve their performance by getting what they cannot observe from the hidden states. Finally, inter-agent communication expands agents’ horizons. By sending high-dimensional embedding vectors, they share their observations with others. With the help of HGAT, agents can cooperate better by using extensive information from those vectors in decision making.

Compared with non-HGAT-based approaches, our method has another advantage in the replay buffer. As they concatenate all local states into a vector and pad 0 for unobserved entities, the space complexity of observations in their replay buffer is O(N×M), where *N* and *M* means the number of agents and entities, respectively. However, our method only stores local states, whose space complexity is O(M). Although it has to store the adjacency matrices $A^{O}$ to represent the relationship among agents, this is more economical than storing observations in terms of storage, as an adjacency matrix represents the relationship between agents by a bit.

## 8. Conclusions

In this paper, we propose a scalable and transferable DRL-based multi-agent coordination control method for cooperative and competitive tasks. This method introduces HGAT, GRU, and inter-agent communication into DRL to improve performance in mixed cooperative–competitive environments. By intensive simulations, our method shows its superiority over DGN, DQN, HAMA, and MADDPG both in UAV recon and predator-prey.

In the future, we will improve our method by introducing an adaptive policy base on the action entropy of the agent to provide a more intelligent exploration. We will evaluate the performance of the entropy-based policy and compare it with the ϵ-greedy and ϵ-categorical policies. Specifically, we will test the capabilities of automating entropy adjustment under different entropy targets in large-scale multi-agent systems. Furthermore, we will try to extend our method into continuous policies and evaluate its performance in cooperative and competitive scenarios with continuous action space.

## Figures and Tables

**Table:** 





**Table:** 





**Table 1:** Experiment parameters of UAV recon.

Parameters	Settings
Target Area	200 × 200
Number of PoIs	120
Recon Range	10
Observation Range	15
Communication Range	30
Maximum Speed	10/s
Energy Consumption for Hovering	0.5
Energy Consumption for Movement	0.5
Penalty Factor *p*	1
Importance Factor $\eta_{1}$	1
Importance Factor $\eta_{2}$	0.1
Timeslots of Each Episode	100

**Table 2:** Experiment parameters of predator-prey.

Parameters	Settings
Target Area	100 × 100
Number of Predators	5
Number of Preys	5
Attack Range	8
Observation Range	30
Communication Range	100
Maximum Speed of Predators	10/s
Maximum Speed of Preys	12/s
Timeslots of Each Episode	100

**Table 3:** The mean and standard deviation of scores in predator-prey.

Predator		Prey	
	**Our Method**	**DGN**	**DQN**
Our method	**0.331**± 0.088	**0.535**± 0.086	**0.591**± 0.101
DGN	0.051 ± 0.060	0.271 ± 0.095	0.386 ± 0.095
DQN	0.014 ± 0.034	0.173 ± 0.086	0.120 ± 0.078
**Predator**		**Prey**	
	**Our Method**	**HAMA**	**MADDPG**
Our method	**0.331**± 0.088	**0.787**± 0.027	**0.472**± 0.098
HAMA	0.051 ± 0.050	0.351 ± 0.050	0.403 ± 0.091
MADDPG	0.038 ± 0.048	0.239 ± 0.090	0.051 ± 0.057

## Data Availability

Not applicable.

## Abbreviations

AC	Actor–Critic
ACO	Ant Colony Optimization
COMA	COunterfactual Multi-Agent
Dec-POMDP	Decentralized Partially Observable Markov Decision Process
DGN	Deep Graph Network
DNN	Deep Neural Network
DQN	Deep Q-Network
DRGN	Deep Recurrent Graph Network
DRL	Deep Reinforcement Learning
GA	Genetic Algorithm
GAT	Graph Attention neTwork
GCN	Graph Convolutional Network
GRU	Gated Recurrent Unit
HAMA	Hierarchical Graph Attention-Based Multi-Agent Actor–Critic
HGAT	Hierarchical Graph Attention neTwork
MAAC	Multi-Actor-Attention-Critic
MADDPG	Multi-Agent Deep Deterministic Policy Gradient
MARL	Multi-Agent Reinforcement Learning
PoI	Point-of-Interest
QoS	Quality of Service
RL	Reinforcement Learning
RSH	Randomized Search Heuristic
SARSA	State-Action-Reward-State-Action
TRPO	Trust Region Policy Optimization
UAV	Unmanned Aerial Vehicle
UAV-MBS	Unmanned Aerial Vehicle Mobile Base Station
VB	Value-Based

## Appendix A. Ablation Study

In the ablation study, we test our method and three variants in UAV recon with 20 UAVs and summarized the performances in Table A1. The variants are described as follows:

- Without H: removing the first HGAT layer;
- Without G: removing GRU in the recurrent unit;
- Without C: disabling inter-agent communication.

**Table A1:** The mean and standard deviation of three metrics in the ablation study (N=20).

Model		Metric	
	**Coverage**	**Fairness**	**Energy Consumption**
Our method	**0.466** ± 0.046	**0.784** ± 0.061	**0.665** ± 0.015
Without H	0.103 ± 0.022	0.696 ± 0.087	0.710 ± 0.011
Without G	0.349 ± 0.065	0.726 ± 0.116	0.730 ± 0.017
Without C	0.412 ± 0.058	0.749 ± 0.081	0.668 ± 0.023
